# Effect of early peri-operative arterial lactate concentration level ratios on post-hepatectomy liver failure

**DOI:** 10.1007/s12672-024-00911-7

**Published:** 2024-03-21

**Authors:** Dong-Dong Wang, Meng-Meng Dong, Ya-Ming Xie, Fei-Qi Xu, Tian-Wei Fu, Yu-Chen Wu, Zhe Zhang, Yi Lu, Lei Liang, Wei-Feng Yao, Guo-Liang Shen, Jun-Wei Liu, Cheng-Wu Zhang, Qiu-Ran Xu, Zun-Qiang Xiao

**Affiliations:** 1 General Surgery, Cancer Center, Department of Hepatobiliary & Pancreatic Surgery and Minimally Invasive Surgery, Zhejiang Provincial People’s Hospital (Affiliated People’s Hospital), Hangzhou Medical College, Hangzhou, Zhejiang China; 2 Jinzhou Medical University Graduate Training Base (Zhejiang Provincial People’s Hospital), Hangzhou, China; 3 MOE Key Laboratory of Macromolecular Synthesis and Functionalization, Department of Polymer Science and Engineering, Zhejiang University, Hangzhou, 310027 China; 4 Zhejiang Key Laboratory of Tumor Molecular Diagnosis and Individualized Medicine, Zhejiang Provincial People’s Hospital, Affiliated People’s Hospital, Hangzhou Medical College, Hangzhou, 310014 Zhejiang China

**Keywords:** Arterial lactate, Hepatocellular carcinoma, Post-hepatectomy liver failure, Severe morbidity, Postoperative haemorrhage

## Abstract

**Background:**

Post-hepatectomy liver failure (PHLF) is a serious complication after hepatectomy and a major cause of death. The current criteria for PHLF diagnosis (ISGLS consensus) require laboratory data of elevated INR level and hyperbilirubinemia on or after postoperative day 5. This study aims to propose a new indicator for the early clinical prediction of PHLF.

**Methods:**

The peri-operative arterial lactate concentration level ratios were derived from time points within the 3 days before surgery and within POD1, the patients were divided into two groups: high lactate ratio group (≥ 1) and low lactate ratio group (< 1). We compared the differences in morbidity rates between the two groups. Utilized logistic regression analysis to identify the risk factors associated with PHLF development and ROC curves to compare the predictive value of lactate ratio and other liver function indicators for PHLF.

**Results:**

A total of 203 patients were enrolled in the study. Overall morbidity and severe morbidity occurred in 64.5 and 12.8 per cent of patients respectively. 39 patients (19.2%) met the criteria for PHLF, including 15 patients (7.4%) with clinically relevant Post-hepatectomy liver failure (CR-PHLF). With a significantly higher incidence of PHLF observed in the lactate ratio ≥ 1 group compared to the lactate ratio < 1 group (n = 34, 26.8% vs. n = 5, 6.6%, *P* < 0.001). Multivariable logistic regression analysis revealed that a lactate ratio ≥ 1 was an independent predictor for PHLF (OR: 3.239, 95% CI 1.097–9.565, *P* = 0.033). Additionally, lactate ratio demonstrated good predictive efficacy for PHLF (AUC = 0.792).

**Conclusions:**

Early assessment of peri-operative arterial lactate concentration level ratios may provide experience in early intervention of complications in patients with hepatocellular carcinoma, which can reduce the likelihood of PHLF occurrence and improve patient prognosis.

## Introduction

Hepatocellular carcinoma (HCC) is a common malignancy and the third leading cause of cancer-related deaths worldwide [1]. Hepatectomy remains the primary treatment option for HCC, but post-hepatectomy liver failure (PHLF), a serious complication, is a common cause of postoperative death [2]. There is an urgent need for more comprehensive and accurate methods to predict the occurrence of PHLF early. Early detection of PHLF can help clinicians identify high-risk patients and take preventive measures to reduce the postoperative hospital stay period and improve the short-term prognosis of patients after hepatectomy.

According to the International Study Group for Liver Surgery (ISGLS), PHLF is defined as the deterioration of two laboratory indicators, namely, the international normalized ratio (INR) and total bilirubin (TBil). Measured on or after postoperative day 5, these parameters reflect the synthesis, excretion, and detoxification functions of the liver [3]. However, using the ISGLS criteria to define post-hepatectomy liver failure has its limitations. Firstly, the definition of PHLF requires the testing of two indicators of coagulation and liver function. Secondly, the impact of preoperative liver function is not taken into consideration. Furthermore, since PHLF with combined elevated INR and hyperbilirubinemia is a postoperative event that is often diagnosed on or after postoperative day 5, its clinical significance as a short-term prognostic factor is relatively limited [4]. This gap of 5 days or more allows for unknown risks to the patient in the interim and delays the treatment of patients with severe liver failure. Although PHLF currently has no definitive treatment, the development of a more convenient diagnostic tool to predict PHLF at an early stage could significantly improve the prognosis of liver resection by allowing for prevention or early intervention [5]. Furthermore, incorporating multiple time points of liver function indicators can provide a more comprehensive assessment of the patient's preoperative liver function and surgical tolerance.

Lactate, a product of tissue metabolism during hypoxia, is normally metabolized by the liver and excreted by the kidneys [6]. Elevated lactate levels are an early sign of impaired tissue microcirculation and serve as an important clinical indicator of not only liver function but also as a biomarker reflecting prognosis [7–9]. Lactate levels are commonly evaluated in acutely ill patients, and lactate level assessments are already being utilized in various fields, including emergency medicine, intensive care, and cardiac surgery [10, 11].In hepatocellular carcinoma, there is evidence supporting the association of lactate levels with overall morbidity and mortality after hepatectomy, but studies related to PHLF are currently limited [6, 12]. However, the prediction of postoperative complications in the liver relies solely on lactate levels before or after surgery. Due to the rapid metabolism of lactate in the body, arterial lactate concentration levels measured at different time points can vary greatly. Therefore, the use of pre- or post-operative measurements of arterial lactate levels at a single time point may not be sufficient to accurately reflect individualized preoperative patient differences or potential interference from intraoperative factors. In general, lactate levels can be obtained rapidly and easily in most clinical settings. Combining pre- and postoperative lactate measurements to assess early peri-operative arterial lactate concentration levels ratios (Postoperative lactate level/preoperative lactate level) could largely reduce the presence of confounding factors, to understand the changing rate of lactate from pre- to postoperative period, and improve the predictability of post-hepatectomy complications.

This study aims to predict the occurrence of PHLF by using early peri-operative arterial lactate concentration level ratios. We aim to compare the ability of different liver function indicators to predict PHLF. Additionally, we aim to investigate the differences in the incidence of other complications between different lactate ratio groups.

## Materials and methods

### Patient selection and data collection

We conducted a retrospective analysis of HCC patients who underwent hepatectomy at Zhejiang Provincial People's Hospital from January 2018 to June 2022. Patients who met the following inclusion criteria were included: (1) HCC patients who underwent hepatectomy, with confirmation of diagnosis through pathological examination of surgical specimens; (2) Child–Pugh A or B; (3) Age above 18 years old. The exclusion criteria were as follows: (1) Presence of biliary obstruction and coagulation disorders; (2) Postoperative hospital stay of fewer than 5 days; (3) Significantly elevated lactate levels were due to severe hypoxia, shock, severe infection or sepsis, and diabetic acidosis; (4) Missing data of important variables used in this study.

All data were obtained from the hospital medical record system. Routine blood tests were performed before and after surgery. From POD5 onwards, we measured TBil and INR levels in patients who were still hospitalized to detect PHLF as defined by the ISGLS. All hematological test variables were uniformly tested in our clinical laboratory. Before surgery, all patients underwent cross-sectional dynamic imaging with electron computed tomography (CT) or magnetic resonance imaging (MRI) to obtain preoperative tumor-related information. Standardized pre- and post-operative laboratory and imaging tests, as well as strict peri-operative management, ensured the validity and accuracy of the test results. This study conformed with the Helsinki Declaration and was approved by the Institutional Review Committee of Zhejiang Provincial People's Hospital. (QT2023142).

### Clinical features and associated variables

The variables included in this study were obtained from the medical record system of Zhejiang Provincial People's Hospital and retrospectively collected. These variables included age, gender, body mass index (BMI), hypertension, cirrhosis, chronic kidney disease, and diabetes mellitus. Surgery-related variables were intraoperative blood loss, intraoperative blood transfusion, the extent of liver resection, inflow occlusion, and operating time. Pre- and post-operative arterial lactate concentration levels (taken at the within 3 days before surgery and within POD1), preoperative prothrombin time (PT), alpha-fetoprotein (AFP), TBil, and INR were also included. Tumor-related variables were obtained from preoperative imaging data, including the number of tumors and maximum tumor size. Patients with preoperative or postoperative histological confirmation of cirrhosis were included, and the extent of liver resection was categorized as minor or major resections in ≥ 3 segments. Inflow occlusion was at the discretion of the surgeon, with clamping for no more than 15 min and relaxation for 5–10 min.

### Definition of peri-operative arterial lactate concentration level ratios and grouping of patients

Previous studies explored the great predictive value of postoperative lactate level for the prognosis of patients after hepatectomy [12]^]^. Based on these, we aimed to assess the extent to which postoperative lactate level plays a role in prognosis while also taking into account the role of preoperative lactate level.

The purpose is to understand the changing rate of lactate from pre- to postoperative period. In the current study, we are trying to investigate the predictive ability of the peri-operative arterial lactate concentration level ratios which is calculated using the formula “Lactate ratio = Postoperative lactate level/preoperative lactate level”. According to the lactate ratio, Patients were divided into two groups: high lactate ratio group (≥ 1) and low lactate ratio group (< 1).

### Definition of PHLF and other complications

The occurrence of the primary outcome of PHLF met the criteria outlined in the ISGLS consensus (Rahbari et al., 2011) [3]. PHLF is defined by an increase in INR levels and hyperbilirubinemia occurring on or after the 5th postoperative day, as measured against local laboratory cut-off values of 24 um/L for TBil and 1.2 mg/dL for INR at Zhejiang Provincial People’s Hospital. The definition of PHLF applies to patients with both normal and abnormal preoperative liver function, regardless of the extent of resection or underlying liver disease. It excludes factors such as biliary obstruction or other pre- and post-operative complications that can cause significant changes in biochemical and clinical parameters. Furthermore, the requirement for coagulation factors, such as fresh frozen plasma (FFP), to maintain normal INR levels in the presence of hyperbilirubinemia on or after postoperative day 5 is also considered indicative of PHLF. In patients with preoperatively increased INR or increased TBil, PHLF is defined by a rising serum bilirubin concentration and increasing INR on or after postoperative day 5 compared with the values of the previous days. In addition the severity of the complication was graded. Grade A PHLF is defined as impairment in these laboratory markers without any therapeutic consequence, grade B implies need of non-invasive treatment and grade C requires ICU monitoring with invasive treatment. Accordingly, PHLF B and C were combined as clinically relevant Post-hepatectomy liver failure (CR-PHLF). Secondary outcomes of interest include overall morbidity, severe morbidity, specific morbidity, mortality within 90 days postoperatively, and days spent in hospital postoperatively. Overall morbidity is defined as the occurrence of one or more events during the peri-operative period, including postoperative haemorrhage, bile leak, surgical site infection, pulmonary infection, and postoperative seroperitoneum. These events are classified as mild (C-D I-II) or severe (C-D IIIa-V) according to the Clavien-Dindo classification.

### Statistical analysis

Data analysis was conducted using IBM SPSS version 25.0 (SPSS Inc.). Patient characteristics were analyzed by comparing continuous variables with the T-test and the Mann–Whitney U test, while categorical variables were assessed using the chi-squared or Fisher's exact test. A univariable logistic regression model was applied to all variables potentially associated with PHLF. Significant variables (*p* < 0.05) in the univariable analysis were used to create a multivariable logistic regression model through a backward stepwise selection process. Odds ratios (OR) and 95% confidence intervals (CI) were calculated to evaluate the strength of the associations between the various factors and PHLF. A *P*-value < 0.05 was considered statistically significant. The receiver operating characteristic (ROC) curve was utilized to assess lactate ratio and the ability of the ROC to differentiate between PHLF and other relevant liver function indicators.

## Results

### Baseline characteristics

**Fig. 1:**
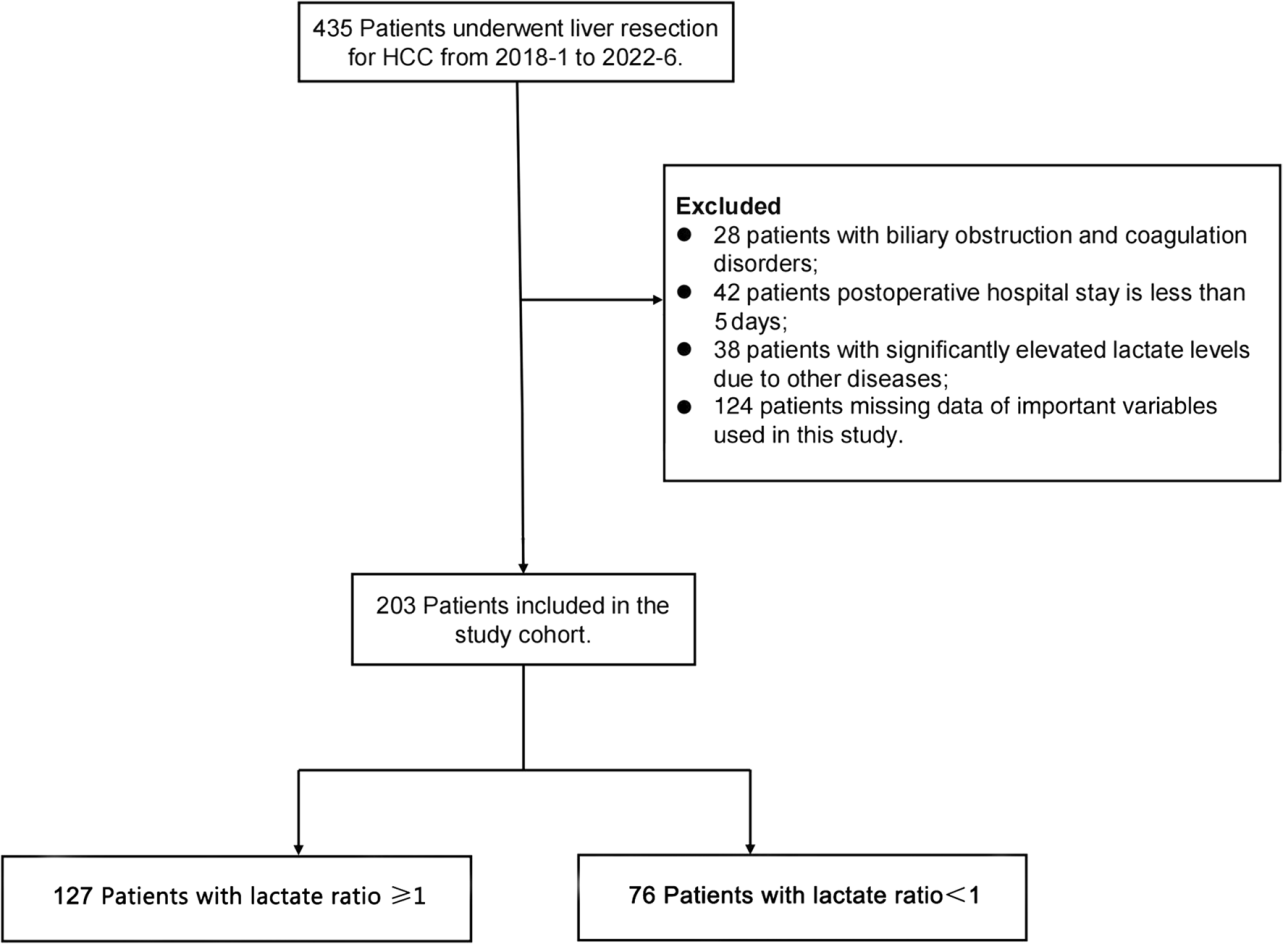
Flow chart of participant population. HCC, Hepatocellular Carcinoma; Lactate ratio, postoperative lactate level/preoperative lactate level

Between January 2018 and June 2022, 435 patients with HCC underwent hepatectomy at Zhejiang ProvincialPeople's Hospital. After screening these patients with sufficient clinical information, we analyzed 203 cases, with PHLF used as the primary outcome endpoint. Of the 203 cases, 127 (62.6%) belonged to the lactate ratio ≥ 1 group, while 76 (37.4%) belonged to the lactate ratio < 1 group. The study flow was presented in Fig. 1. Out of all 203 patients, 170 (83.7%) were male, with a mean age of 62 years. Cirrhosis was present in 72.9% of the patients. Only a minority of patients have concomitant chronic kidney disease (2.5%). There were no significant differences (*p* > 0.05) in most of the baseline variables of interest in terms of disease-related or surgical variables, except for cirrhosis, intraoperative blood loss, intraoperative blood transfusion, operating time, AFP, and TBil, as shown in Table 1.

**Table 1:** Comparison of the clinical characteristics of the two groups

Variable	Total (n = 203)	Lactate ratio ≥ 1 (n = 127)	Lactate ratio < 1 (n = 76)	*P*-value
Age^a^	62 (54–69)	61 (54–67)	63.5 (55.25–70)	0.070
Male	170 (83.7%)	111 (87.4%)	59 (77.6%)	0.068
BMI ^a^	22.95 (21.05–25.22)	22.67 (21.19–25.28)	23.37 (20.27–25.20)	0.914
Hypertension	81 (39.9%)	51 (40.2%)	30 (39.5%)	0.923
Cirrhosis	148 (72.9%)	99 (78.0%)	49 (64.5%)	**0.037**
Chronic kidney disease	5 (2.5%)	2 (1.6%)	3 (3.9%)	0.557
Diabetes mellitus	25 (12.3%)	14 (11.0%)	11 (14.5%)	0.469
Intraoperative blood loss^a^	300 (100–650)	400 (200–800)	200 (100–500)	**0.001**
Intraoperative blood transfusion	69 (34.0%)	50 (39.4%)	19 (25.0%)	**0.036**
Extent of liver resection ≥ 3 segments	53 (26.1%)	38 (29.9%)	15 (19.7%)	0.110
Inflow occlusion	80 (39.4%)	51 (40.2%)	29 (38.2%)	0.778
Operating time^a^	235 (165–295)	240 (180–310)	200 (150–268.75)	**0.001**
PT^a,b^	11.9 (11.40–12.72)	12 (11.40–12.70)	11.9 (11.10–12.90)	0.650
AFP^a,b^	32.7 (4.80–838.30)	94.1 (7.10–968.40)	10.5 (3.80–402.35)	**0.007**
TBil^a,b^	15.9 (12.40–19.60)	16.6 (13.60–20.80)	14.55 (10.15–17.68)	**0.002**
Maximum tumor size > 5 cm	83 (40.9%)	56 (44.1%)	27 (35.5%)	0.229
Multiple tumors	36 (17.7%)	24 (18.9%)	12 (15.8%)	0.575

Values in parentheses are percentages unless indicated otherwise; ^a^Values are median (range); ^b^Pre-operative laboratory indicators

The bold values indicate statistical signifcance (*P* < 0.05)

BMI, body mass index; PT, prothrombin time; AFP, alpha-fetoprotein; TBil, total bilirubin; Lactate ratio, postoperative lactate level/preoperative lactate level

### Independent risk predictive factors for post-hepatectomy liver failure

In this study, we screened ten potential risk factors for PHLF through a univariable logistic regression analysis; these included cirrhosis, intraoperative blood loss, intraoperative blood transfusion, extent of liver resection, inflow occlusion, operating time, lactate ratio, PT, AFP, and TBil. Variables with unadjusted *P* values < 0.05 were included in the multivariable logistic regression analysis, and six variables were identified for the final model. Our results revealed that lactate ratio ≥ 1 were significantly associated with PHLF events and was considered an excellent independent predictor of PHLF (OR: 3.239, 95% CI 1.097–9.565, *p* = 0.033). Furthermore, multivariable logistic regression analysis revealed that cirrhosis (OR: 4.929, 95% CI 1.085–22.400, *p* = 0.039), intraoperative blood loss  ≥ 800 ml (OR: 2.705, 95% CI 1.152–6.350, *p* = 0.022), operative time  ≥ 240 min (OR: 2.832, 95% CI 1.180–6.795, *p* = 0.020), PT  ≥ 13s (OR: 3.189, 95%CI 1.291–7.880, *p* = 0.012) and TBil ≥ 17umol/L (OR: 2.440, 95%CI 1.059–5.624, *p* = 0.036) were also independently associated with the occurrence of PHLF. The independent risk factors for PHLF are shown in Table 2**.**

**Table 2:** Univariable and multivariable logistic regression analysis of factors associated with post-hepatectomy liver failure

Variables	OR Comparison	UV OR (95%CI)	UV *P*-value^a^	MV OR (95%CI)	MV *P*-value
Age	≥ 65 vs. < 65 years	0.502 (0.238–1.059)	0.070		
Gender	male vs. Female	2.687 (0.775–9.307)	0.119		
BMI	≥ 25 vs. < 25 kg/m^2^	0.529 (0.218–1.280)	0.158		
Hypertension	Yes vs. No	0.528 (0.246–1.131)	0.100		
Cirrhosis	Yes vs. No	8.833 (2.051–38.037)	0.003	**4.929 (1.085–22.400)**	**0.039**
Chronic kidney disease	Yes vs. No	1.053 (0.114–9.689)	0.964		
Diabetes mellitus	Yes vs. No	0.538 (0.152–1.897)	0.335		
Intraoperative blood loss	≥ 800 vs. < 800 ml	4.163 (1.968–8.809)	< 0.001	**2.705 (1.152–6.350)**	**0.022**
Intraoperative blood transfusion	Yes vs. No	3.687 (1.789–7.601)	< 0.001	NA	0.297
Extent of liver resection	≥ 3 vs. < 3	3.632 (1.746–7.557)	0.001	NA	0.239
Inflow occlusion	Yes vs. No	2.076 (1.025–4.205)	0.042	NA	0.281
Operating time	≥ 240 vs. < 240 min	3.685 (1.717–7.910)	0.001	**2.832 (1.180–6.795)**	**0.020**
Lactate ratio	≥ 1 vs. < 1	5.191 (1.932–13.947)	0.001	**3.239 (1.097–9.565)**	**0.033**
PT	≥ 13 vs. < 13 s	2.909 (1.361–6.219)	0.006	**3.189 (1.291–7.880)**	**0.012**
AFP	≥ 400 vs. < 400 ug/L	2.512 (1.228–5.138)	0.012	NA	0.213
TBil	≥ 17 vs. < 17 uml/L	3.444 (1.660–7.143)	0.001	**2.440 (1.059–5.624)**	**0.036**
Maximum tumor size	> 5 vs. ≤ 5 cm	1.919(0.949–3.882)	0.070		
Tumor number	Multiple vs. Solitary	0.812(0.312–2.112)	0.670		

The bold values indicate that these variables were statistically significant in the multivariable analyses (*P* < 0.05)

^a^Variables found to be significant at *P* < 0.05 in univariable analyses were entered into multivariable logistic regression analyses

BMI, body mass index; PT, prothrombin time; AFP, alpha-fetoprotein; TBil, total bilirubin; OR, odds ratio; UV, univariable; MV, multivariable; NA, not available; Lactate ratio, postoperative lactate level/preoperative lactate level

### The predictive power of lactate and other liver function indicators for post-hepatectomy liver failure

**Fig. 2:**
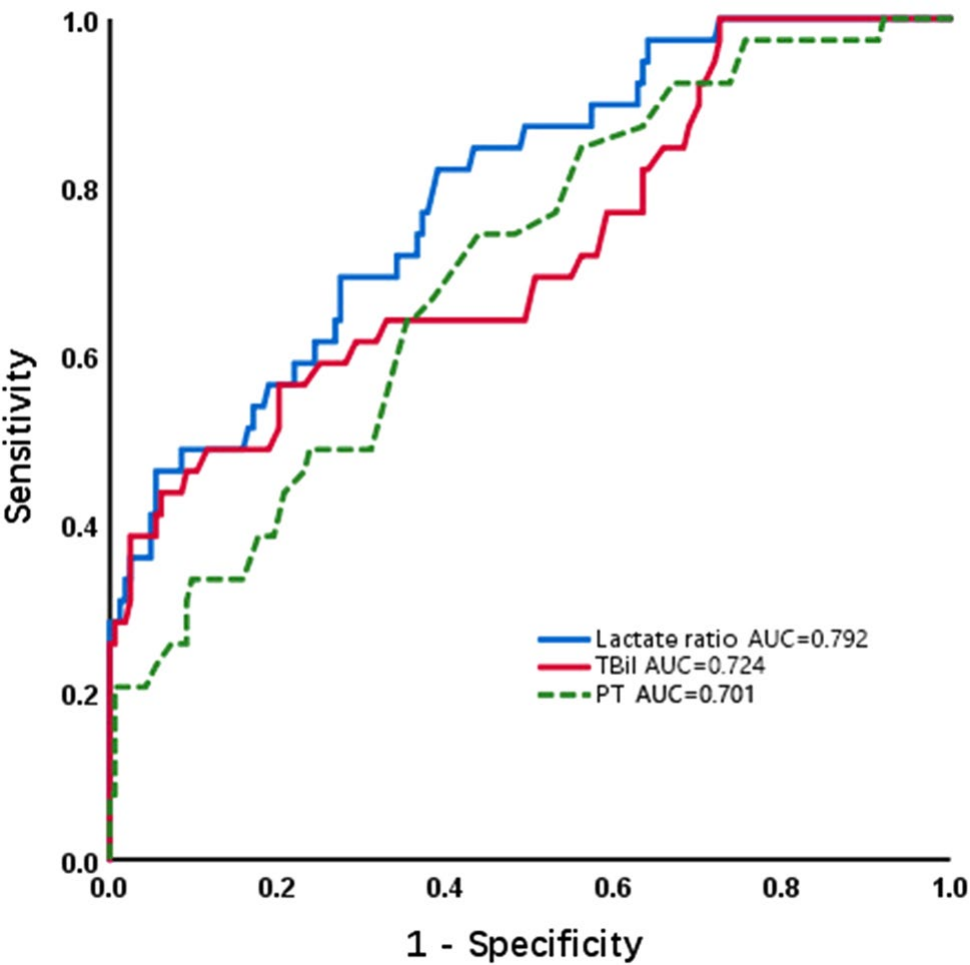
Comparison of liver function indicators for predicting post-hepatectomy liver failure by using ROC curves. ROC, Receiver Operating Characteristic; AUC, Area Under Curve; TBil, total bilirubin; PT, prothrombin time; Lactate ratio, postoperative lactate level/preoperative lactate level

In view of the impact of preoperative liver function status on post-hepatectomy prognosis, it is important to evaluate liver function using preoperative TBil and PT. Additionally, risk factors such as lactate ratio, TBil, and PT are independently associated with PHLF through multivariable logistic regression analysis. ROC curves were constructed to further assess the discrimination ability of these factors. Lactate ratio, TBil, and PT were used to predict the ability to develop PHLF, as shown in Fig. 2. The discrimination ability of lactate ratio, as assessed by the AUC, was 0.792 (95% CI 0.715–0.869; *p* < 0.001). However, the AUC value of TBil was 0.724 (95% CI 0.629–0.819; *p* < 0.001), and the AUC value of PT was 0.701 (95% CI 0.613–0.789; *p* < 0.001). These results indicate that the lactate ratio have a higher discrimination ability than other indicators.

### Association of lactate levels with post-hepatectomy liver failure and other complications

**Fig. 3:**
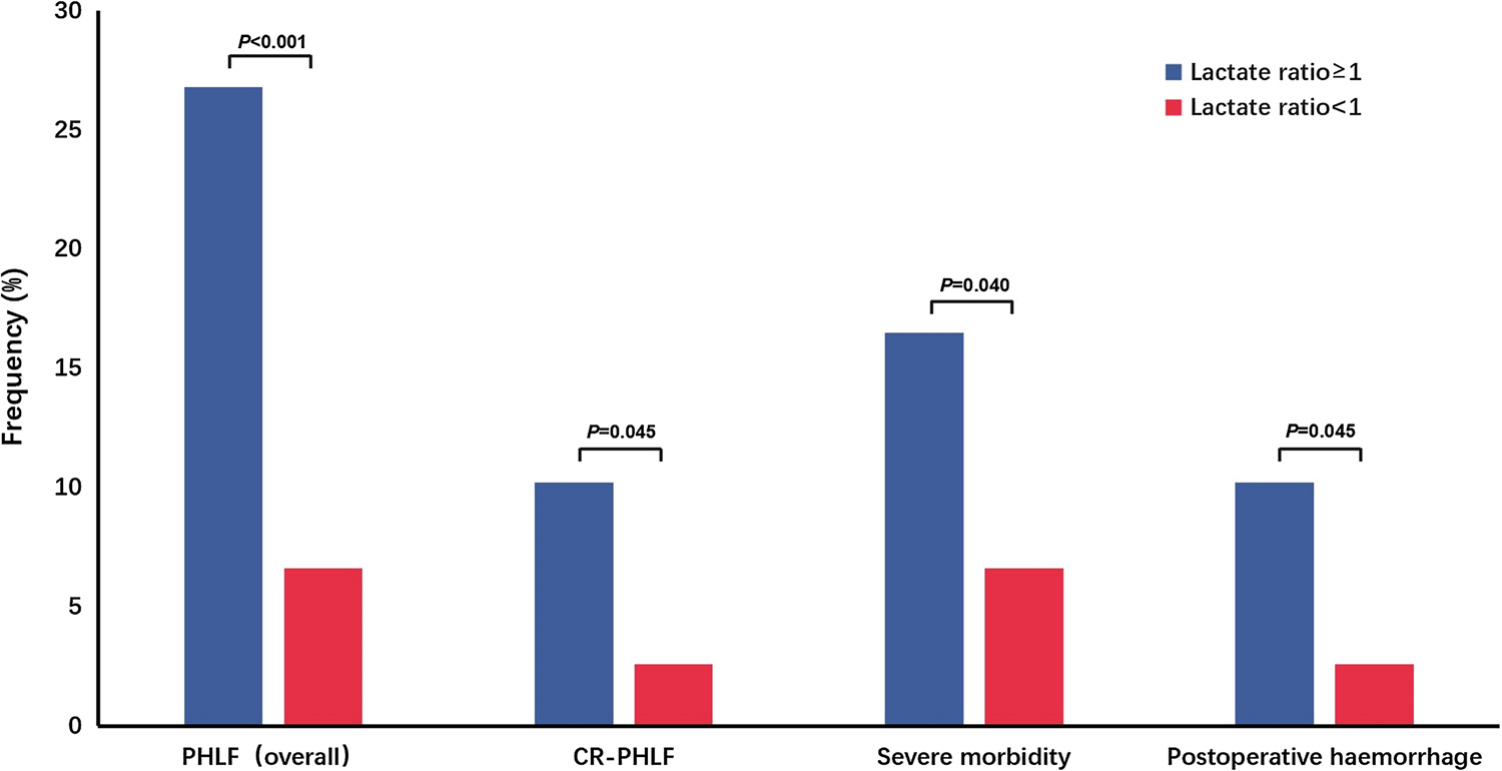
The differences in complications among different lactate ratio groups were compared. *P* < 0.05 was considered statistically significant. PHLF, Post-hepatectomy liver failure; CR-PHLF, Clinically relevant Post-hepatectomy liver failure; Lactate ratio, postoperative lactate level/preoperative lactate level

Table 3 and Fig. 3 depict the distribution of lactate ratio in relation to morbidity in two different groups. In terms of the primary outcome, 39 patients were diagnosed with PHLF, and the incidence of PHLF was significantly higher in the lactate ratio ≥ 1 group compared to the lactat ratio < 1 group (n = 34, 26.8% vs. n = 5, 6.6%, *p* < 0.001). Among the patients with PHLF, CR-PHLF was achieved in 15 patients, and there was a significant difference in the incidence of CR- PHLF between the two lactate level ratio groups (n = 13, 10.2% vs. n = 2, 2.6%, *p* = 0.045). Additionally, the incidence of morbidity was higher in the lactate ratio ≥ 1 group, except for postoperative hospital stay. Furthermore, there were significant differences between the two groups in the incidence of severe morbidity (n = 21, 16.5% vs. n = 5, 6.6%, *p* = 0.040) and postoperative haemorrhage (n = 13, 10.2% vs. n = 2, 2.6%, *p* = 0.045), with comparable results.

**Table 3:** The differences in complications among different lactate ratio groups were compared

Complications	Overall (n = 203)	Lactate ratio ≥ 1 (n = 127)	Lactate ratio < 1 (n = 76)	p Value
PHLF overall	39 (19.2%)	34 (26.8%)	5 (6.6%)	**< 0.001**
CR-PHLF	15 (7.4%)	13 (10.2%)	2 (2.6%)	**0.045**
Morbidity (overall)	131 (64.5%)	84 (66.1%)	47 (61.8%)	0.535
Morbidity (C-D IIIa-V)	26 (12.8%)	21 (16.5%)	5 (6.6%)	**0.040**
90-d mortality	5 (2.5%)	5 (4.0%)	0 (0%)	0.192
Postoperative hospital stay (days)*	9 (7–12)	9 (7–12)	9 (7–12)	0.807
Specific morbidity				
Postoperative haemorrhage	15 (7.4%)	13 (10.2%)	2 (2.6%)	**0.045**
Bile leakage	10 (4.9%)	7 (5.5%)	3 (3.9%)	0.870
Surgical site infection	11 (5.4%)	8 (6.3%)	3 (3.9%)	0.692
Pulmonary infection	32 (15.8%)	21 (16.5%)	11 (14.5%)	0.696
Seroperitoneum	59 (29.1%)	42 (33.1%)	17 (22.4%)	0.104

Values in parentheses are percentages unless indicated otherwise; *Values are median (range)

The bold values indicate statistical signifcance (*P* < 0.05)

PHLF, Post-hepatectomy liver failure; CR-PHLF, Clinically relevant Post-hepatectomy liver failure; C–D, Clavien–Dindo classification; Lactate ratio, postoperative lactate level/preoperative lactate level

### Lactate levels stratify the risk level of patients with post-hepatectomy liver failure

We stratified the pre- and post-operative lactate levels to demonstrate the risk of PHLF in high-risk patients. The normal lactate level of 1.6 mmol/L was used as the cut-off value in our hospital. The patients were divided into three groups: elevated pre-and post-operative lactate levels of ≥ 1.6 mmol/L (both elevated), normal pre-and post-operative lactate levels of < 1.6 mmol/L (both normal), and elevated postoperative lactate level of ≥ 1.6 mmol/L (only postoperative elevated). Our analysis revealed a significantly higher incidence of PHLF in the group with elevated lactate pre-and post-operatively and the group with only postoperative elevated lactate, compared to patients with both normal lactates (n = 2, 5.1% vs. n = 25, 64.1%, *p* = 0.002 and n = 2, 5.1% vs. n = 36, 92.3%, *p* = 0.003). There were also significant differences in the occurrence of PHLF within the groups, As shown in Table 4**.**

**Table 4:** Pre- and post-operative lactate levels stratify patients with post-hepatectomy liver failure

Groups	Total (n = 203)	PHLF (n = 39)	Non-PHLF (n = 164)	*P*-value
Both elevated	87 (42.9%)	25 (64.1%)*	62 (37.8%)	**0.003**
Only postoperative elevated	133 (65.5%)	36 (92.3%)**	97 (59.1%)	**< 0.001**
Both normal	40 (19.7%)	2 (5.1%)	38 (23.2%)	**0.011**

The bold values indicate statistical signifcance (*P* < 0.05)

*Compared to the group with both normal, *p* = 0.002; **Compared to the group with both normal,* p* = 0.003

Values in parentheses are percentages; PHLF, Post-hepatectomy liver failure

## Discussion

Post-hepatectomy liver failure (PHLF) is a serious condition that indicates impaired liver function and is strongly associated with poor prognosis [13]. The “50–50” criteria and MELD score limit the prediction of PHLF due to their time lag and complexity [14, 15]. Early identification and intervention are critical for improving patient outcomes. Early intervention can not only reduce the occurrence of postoperative complications such as liver dysfunction, bleeding, infection, and hepatic encephalopathy caused by PHLF but also reduce the risk of postoperative deterioration and death [16, 17]. Early intervention of PHLF can also promote the regeneration and repair of hepatocytes and accelerate the recovery of liver function after surgery with the aid of reasonable nutritional support and drug intervention [18]. In addition, it can help patients recover their liver function more quickly and reduce the length of hospital stay. To address the limitations of current detection tools, we attempted to combine variables from the early peri-operative phase to create criteria that predicted the occurrence of PHLF more accurately than existing definitions.

As the liver is the main site of lactate metabolism, it becomes a source of high lactate production when damaged. Hyperlactatemia occurs frequently following liver resection for various reasons, such as specific intraoperative techniques [19], anesthesia methods, and treatments adopted in liver resection surgery, including the need to maintain a low central venous pressure, the blockage of the first hepatic portal, the degree of liver tissue resection during the operation, a hypoxic microenvironment, and a state of hypoperfusion created in the liver. Hepatic ischemia itself leads to the release of cytokines, and the patient’s liver function is transiently impaired, causing an increase in lactate levels [20, 21]. Blood lactate is a sensitive indicator of instantaneous liver function; due to its timeliness and sensitivity, it is also ideal for short-term assessment of the state of liver damage [22]. The current studies on PHLF prediction only consider the influence of intraoperative factors [23]. Even though preoperative fluctuations in lactate are relatively small, the impact of preoperative liver function on the occurrence of PHLF in patients should not be ignored. The early peri-operative arterial lactate concentration level ratios integrates the effects of preoperative liver function and intraoperative factors. It not only reflects the severity of postoperative liver function impairment and deterioration in patients with hepatocellular carcinoma in several aspects but also excludes the possibility of a transient elevation of lactate due to significant abnormalities in liver function at a single time point [24], thus eliminating interfering factors to a greater extent.

In the present study, we predicted the occurrence of PHLF by a comprehensive assessment of early peri-operative arterial lactate concentration levels. We found that elevated lactate ratio (≥ 1) were an independent risk factor for the development of PHLF in patients with hepatocellular carcinoma. Ideally, liver function indicators would show a decreasing trend during the early postoperative period. However, due to the surgical approach, the impaired physical condition of the postoperative patient, and severe postoperative stress, higher levels of lactate concentration often occur after hepatectomy [6, 25]. Even if some patients do not have a preoperative disease that causes elevated lactate levels, patients with hepatocellular carcinoma may have varying degrees of concomitant impaired liver function which prevent preoperative lactate level from reaching normal levels. Due to the significant influence of surgical factors, preoperative lactate level fluctuate significantly less than the postoperative lactate level between individual patients. This leads to an increase in the Postoperative lactate level/preoperative lactate level.

We also have found that several factors, such as a history of cirrhosis, intraoperative blood loss, operative time, prolonged PT, and TBil are independent predictors of PHLF, which is consistent with previous studies [23]. Complex hepatectomies are particularly susceptible to a range of factors causing tissue hypoxia; therefore, prevention of intraoperative blood loss and transfusion are crucial to prevent liver failure [26, 27]. Some studies have also found a higher mortality rate in HCC patients with a history of diabetes secondary to PHLF, possibly due to altered liver metabolism, decreased immune function, and hepatic steatosis [28]. However, accurate preoperative evaluation of the patient can minimize the risk of PHLF associated with preoperative diabetes. Inflow occlusion, used to reduce intraoperative bleeding, is also often considered a risk factor for PHLF mainly due to hepatic ischemia and reperfusion injury caused by pedicle clamping [29]. In our study, even though inflow occlusion was associated with PHLF in the univariable analysis, it was not independently associated with the development of PHLF in a multivariable logistic regression analysis, which is consistent with recent reports [23, 30]. This may be closely related to the timing, the mode, and the site of the inflow occlusion. The majority of patients in our sample had good preoperative liver function levels and underwent minor hepatectomies, resulting in a short median inflow occlusion time. The previous study stated that if the maximum tumour size is too large (> 10 cm), the more extensive the liver resection (greater than half hepatectomy), so as to ensure that the resection margins of the tumour are negative. This will greatly increase the risk of PHLF [31, 32]**.** Patients can suffer from persistent hyperbilirubinemia and coagulation disorders due to poor preoperative liver function and intraoperative factors. Meanwhile, preoperative TBil and PT levels are also considered to be important preoperative predictors of the prognosis in liver surgery [26, 33]. Finally, we found that the post- and pre-operative lactate level ratios has a higher area under the curve (AUC) value than other markers (0.792; *p* < 0.001), indicating its better predictive ability for PHLF. Combining lactate measurements at different time points further enhances its predictive advantage.

The detrimental impact of early peri-operative arterial lactate concentration level were associated with severe postoperative morbidity at 30 days, increased 90-day mortality, and a longer length of postoperative hospital stay [25]. However, in some cases, the increased postoperative hospital stay may be due to better management of the patient's disease and monitoring of the recovery process, rather than surgical failure or poor prognosis. The patient population with 90-day mortality was observed in the lactate ratio ≥ 1group, but the sample size limitations rendered the results statistically insignificant. This indicates that a larger sample size is needed for validation. Similar to the occurrence of PHLF, the occurrence of other complications is closely linked to the patient's poorer nutritional status postoperatively and the decline in protein synthesis, coagulation, and detoxification caused by deteriorating liver function. Although our prediction of postoperative complications using early post- and pre-operative arterial lactate concentration level ratios is similar to the outcome predicted by lactate taken at a single time point, we believe that considering the early peri-operative arterial lactate concentration levels will enable a more accurate prediction of postoperative complications as it considers both preoperative liver function and intraoperative factors.

The occurrence of PHLF and other complications in patients with hepatocellular carcinoma can be attributed to multiple factors. Therefore, prevention should be the primary focus, with emphasis on fine intraoperative actions and close monitoring of liver function before and after surgery to facilitate early recognition and timely treatment. Furthermore, Since we are the first study to use lactate ratio to predict PHLF, a single lactate measurement on admission or after surgery should not be used as the sole criterion when assessing liver function in surgical patients. Classifying the severity of PHLF occurrence in patients before obtaining a definitive diagnosis of PHLF allows for more precise prevention and avoids excessive waste of medical resources. Early intervention should be implemented in high-risk patients with a significant increase in lactate ratio and abnormal lactate levels in all cases to prevent the development of multi-organ failure caused by PHLF. However, there is no clear evidence base or consensus guidelines on acceptable lactate ratio cut-off levels and the use of early peri-operative arterial lactate concentration levels to predict the occurrence of complications after hepatectomy. Nevertheless, due to the clinical accessibility of lactate, it is recommended that the early peri-operative arterial lactate concentration level ratios be included as a prognostic reference as it may provide informative significance in predicting the occurrence of PHLF.

The primary limitations of this study is that are the relatively small sample size, observational retrospective study, and no more exploration of the CR-PHLF classification in the ISGLS consensus. To address these limitations, a multicenter cohort study of patients is necessary to explore the value in CR-PHLF. Additional laboratory indicators of liver function are necessary to further validate our results. Future research may be directed toward combining lactate levels with other indicators of liver function.

## Conclusions

In conclusion, our study established that an elevated early peri-operative arterial lactate concentration level ratio in patients has a high predictive value for the incidence of PHLF. It also plays a crucial role in predicting the development of other complications. Therefore, early peri-operative arterial lactate concentration level ratios can be utilized as a valid biomarker to predict prognosis and guide further treatment after hepatectomy.

## Data Availability

The datasets generated during and/or analysed during the current study are available from the corresponding author on reasonable request.
